# Zero-shot traffic flow prediction with foundation models: a comparison with deep learning approaches

**DOI:** 10.1038/s41598-026-50464-8

**Published:** 2026-05-04

**Authors:** Yue Li, Qunshan Zhao, Mingshu Wang

**Affiliations:** 1 Urban Big Data Centre, School of Social and Political Sciences, University of Glasgow, 7 Lilybank Gardens, Glasgow, G12 8RZ UK; 2 School of Geographical and Earth Sciences, University of Glasgow, 7 Lilybank Gardens, Glasgow, G12 8RZ UK

**Keywords:** Traffic flows, Time-series prediction, Deep learning, Foundation models, Engineering, Mathematics and computing

## Abstract

Traffic flow prediction plays an important role in managing urban transportation systems, helping to reduce congestion and improve road safety. Although existing deep learning models improve their predictive accuracy with complex architectures, they require large datasets for task-specific training. Recently, the rapidly developed foundation models have shown outstanding performance in time series prediction. Motivated by the development, we apply two foundation models, Lag-Llama and Chronos, for zero-shot traffic flow prediction and compare their accuracy with that of deep learning models. Our results show that foundation models outperform deep learning models in traffic flow prediction under both normal conditions and disruptive events. Unlike deep learning models, which require large-scale historical data and extensive training time for each task, pre-trained foundation models can be directly applied to datasets with varying data sizes, traffic dynamics, and context lengths. We also find that foundation models with longer context lengths and larger model sizes achieve higher prediction accuracy but require increased inference times. Selecting an appropriate foundation model is also crucial—models trained on a comprehensive dataset are more likely to achieve superior zero-shot performance, making them a practical and efficient choice for real-world traffic prediction applications.

## Introduction

Traffic flow prediction is a critical task of intelligent transportation systems (ITS)^1,2^, focusing on predicting future traffic flow conditions based on historical and real-time data^3–6^. Accurate traffic prediction plays a crucial role in urban planning, infrastructure management, and traffic control, which helps to reduce traffic congestion, enhance road safety, and lower environmental impacts^6–9^. Due to the rapid growth of urban populations and vehicle ownership worldwide, cities face increasing challenges to maintain smooth traffic flow^10^. Effective prediction of traffic flows provides transportation authorities, city planners, and travellers with timely information that enables better decision-making, more efficient resource allocation, and an improved quality of life^2,11,12^.

The traffic flow prediction models have evolved from traditional statistical approaches to deep learning models in recent years^5^. Initially, statistical models such as Autoregressive Integrated Moving Average (ARIMA) dominated the field due to their simplicity, interpretability^13^, and effectiveness in modelling linear^14^ and stationary time-series data. However, these models often struggled to capture complex, non-linear relationships in real-world traffic conditions^15^. To overcome these limitations, deep learning models have been applied broadly, including Convolutional Neural Networks (CNNs), Long Short-Term Memory (LSTM) networks, and more recently, Transformer-based architectures^2,6,15–18^. CNNs are good at capturing local temporal patterns in traffic data by applying convolution operations along the time dimension^19^, while LSTMs are effective in modelling long-term temporal dependencies because of their gated mechanisms^20–22^. Transformer-based models further improve on prediction accuracy by using self-attention mechanisms^18^ to capture both short- and long-term dependencies in traffic data more efficiently and flexibly. Despite their improved accuracy and adaptability to complex scenarios, deep learning models typically require extensive computational resources and large datasets for training^11^, which presents challenges for practical implementation and real-time applications. Besides, these models are often developed for specific tasks^23^, which may lead to overfitting and reducing their ability to generalise effectively to new or unseen data.

In the context of government mobility interventions and large-scale disruptive events, such as COVID-19, traffic flow prediction becomes even more challenging. The imposed restrictions, such as lockdowns and social distancing measures, caused rapid changes in travel behaviour, leading to increased irregularities and deviations from historical traffic trends^24–30^. Most existing traffic prediction models are trained only on pre-pandemic data^12,13,22,31^, which limits their ability to predict traffic flows during periods of significant disruption accurately. Limited models have attempted to address this challenge by considering external factors, such as the effects of imposed response measure^32^ and the evolving status of COVID-19^33^. Another study decomposed irregular traffic flows into distinct attributes and predicted them separately to improve model performance during the pandemic^34^. However, these models often rely on extensive, labeled datasets that capture the effects of mobility restrictions and event status over time, making them difficult to implement in real time and across regions with differing policies.

Recently, the development of Large Language Models (LLMs) has stimulated interest in developing ‘foundation models’ for time serie^23^ and introduced new opportunities for traffic flow prediction^5,35^. Initially designed for natural language processing tasks, LLMs learn extensive general knowledge by pre-training on large amounts of textual data^36–38^. A distinctive strength of pre-trained LLMs is their ability to perform zero-shot prediction, enabling them to perform various tasks without requiring task-specific training examples^39–41^. Models such as GPT have demonstrated impressive zero-shot performance across numerous language understanding and generation tasks from different domains^36,42–44^. Motivated by these advances, researchers have started exploring foundation models for time series prediction^40,41,45^. By specifically pre-training existing transformer-based model architectures on large-scale time-series datasets, these models learn to effectively capture temporal patterns and dynamics in sequential data^23,46^. Inspired by this capability, we are motivated to apply time-series foundation models to zero-shot traffic flow prediction tasks, enabling accurate forecasting of traffic conditions without the need for large amounts of task-specific data.

The overarching goal of this research is to present a comprehensive analysis comparing the performance of deep learning models and time-series foundation models for traffic flow prediction under normal conditions and during disruptive events. The contribution of this paper is threefold. First, it bridges the research gap in applying foundation models to traffic flow prediction by comparing the performance of two groups of time series foundation models and deep learning models on the SCOOT dataset^47^. Second, it evaluates prediction accuracy on heterogeneous and unusual traffic patterns, an area that has been sparsely explored in previous research. It utilises a long-term traffic flow dataset that includes a unique global pandemic period, which allows models to capture long-term traffic trends, seasonal fluctuations, and emergency-related variations, contributing to more robust predictive performance. Third, it highlights the performance gap between time-series foundation models with different model sizes and the diversity and temporal coverage of their training data. A well-trained foundation model with comprehensive datasets is more likely to achieve superior zero-shot performance, making it a practical and efficient choice for real-world traffic flow prediction applications. The research outputs will support city planners in integrating time-series foundation models into intelligent traffic control systems, thereby enhancing their ability to respond effectively to both routine traffic conditions and unexpected disruptions. It is valuable in helping transportation authorities and urban policymakers make informed, data-driven decisions in traffic management for future large-scale emergencies.

## Background

### Traffic flow prediction

Traffic prediction aims to forecast key factors such as vehicle flow, speed, and congestion levels^48–54^. Traffic flow prediction is one of the most fundamental and widely studied tasks in ITS. Traditional approaches typically rely on statistical models, such as ARIMA^55^ or Kalman filter^56^, to capture traffic patterns and seasonalities, often serving as a strong baseline when data exhibit relatively stable trends^14^. While these models are relatively straightforward and interpretable^13^, they may struggle with irregular fluctuations in large-scale transportation networks^15^. To address these complexities, researchers introduced machine learning models such as Random Forests^57^ and Support Vector Machines^58^. By integrating a richer set of input features, these methods can account for additional factors like weather conditions, special events, or road incidents^59^. Although more flexible than purely statistical techniques, they often struggle to achieve consistently robust performance across diverse traffic scenarios.

In recent years, deep learning approaches have shown significant promise due to their ability to automatically extract features and handle complex dependencies. Convolutional Neural Networks (CNNs), traditionally used for image data, have been adapted for time series prediction by applying convolutional filters along the temporal dimension^19^. This allows CNNs to extract local features, detect short-term patterns, and reduce noise in traffic data. Recurrent Neural Networks (RNNs) further enhance sequence modelling by processing time series data, capturing the dynamic behaviour of traffic flow^60–62^. To overcome limitations such as vanishing gradients in standard RNNs, Long Short-Term Memory (LSTM) networks, an advanced type of RNN, employ gating mechanisms to maintain long-term dependencies^63–67^, making them especially effective for predicting complex temporal patterns, such as rush-hour peaks or irregular traffic flows.

More recently, transformer-based models have gained attention for traffic flow prediction due to their self-attention mechanism^68^, which enable efficient modelling of long-term temporal dependencies and global interactions without relying on recurrent structures. By capturing both short- and long-term patterns in parallel, transformers have demonstrated strong performance and scalability in large-scale traffic prediction tasks. Among these models, Informe^18^ is a representative transformer-based architecture for long-sequence time series forecasting that improves computational efficiency through a probabilistic, sparse self-attention mechanism while maintaining the ability to capture long-term temporal dependencies.

### Large language models

Recent progress in computer hardware and the availability of large text datasets have led to the development of transformer-based LLMs that demonstrate impressive performance on various natural language processing tasks^11,37,69,70^. Language models are designed to predict the next token in a sequence by estimating the probability of each token based on those that have already appeared^23^. Tokens may be characters, subwords^71^, or words from a vocabulary. The transformer architecture^72^ was initially developed as an encoder-decoder system for machine translation^23^ and is currently applied in many popular models, such as BART^73^ and T5^74^. In these models, the input text is first converted into a continuous representation using an encoder, after which the decoder generates output tokens sequentially based on the representation and previous tokens. Alternatively, a decoder-only architecture, used in models like GPT-3^36^ and Llama 2^69^, only considers tokens before the current token when making predictions. This architecture simplifies the model’s design while still achieving robust performance.

LLMs are trained on extensive collections of text and can have millions to hundreds of billions of parameters ^74,75^. Researchers have found that increasing the number of parameters in these models leads to better performance ^36^. When the number of parameters becomes large enough, LLMs perform traditional language tasks more accurately and show new abilities that smaller models lack^11^. Zero-shot generalisation is one such ability where the model makes predictions on tasks it was not explicitly trained for^23^. For example, Brown et al*.*^36^ demonstrated that as the number of parameters increases, LLMs acquire the ability to handle new tasks without additional task-specific training. This connection between model parameters and zero-shot generalisation highlights that LLMs not only improve their flexibility and power in language understanding and generation but also become capable of tackling challenges such as forecasting time-series data.

### Large language models for time-series prediction

LLMs have recently developed as useful tools for time series forecasting by using their powerful sequence modelling and pattern recognition capabilities^39^. PromptCast^45^ first treats time series forecasting as a natural language generation task, converting numerical inputs and outputs into textual prompts, thus allowing general-purpose language models to serve as core forecasting engines. However, PromptCast often requires carefully designed prompts, which can be time-consuming in complex or domain-specific scenarios. LLMTime^40^ addresses these limitations by directly tokenising time-series data and treating forecasting as next-token prediction. This tokenisation strategy not only avoids extensive prompt engineering but also enables pre-trained LLMs, such as GPT-3 and LLaMA, to produce robust zero-shot forecasts across a variety of benchmark datasets^23,40^. However, LLMTime can be computationally and memory-intensive due to the large size of the models, and it requires careful data rescaling to handle varying magnitudes and precision.

### Time series foundation models

Unlike PromptCast and LLMTime, which repurpose large pre-trained LLMs with textual or digit-based prompts, researchers have further developed time series foundation models by training them on large, diverse time series datasets. Rasul et al*.* (2024) propose a foundation model (Lag-Llama) designed explicitly for univariate time series forecasting. Built on a decoder-only transformer architecture that uses lag features as covariates, Lag-Llama is pre-trained on a broad collection of real-world time series across multiple domains, including energy, transportation, economics, environmental science, air quality and cloud operations. This large-scale pre-training process enables it to learn a wide range of time-series patterns, resulting in strong zero-shot generalisation performance. Recent concurrent work, Chronos^23^, offers a similarly broad framework for pretrained time series forecasting but adapts transformer-based language model architectures T5^74^ to treat real-valued time series as discrete tokens. Using scaling and uniform binning, Chronos converts continuous sequences into a fixed vocabulary. Once tokenised, it trains a language model on an extensive collection of public and synthetic time-series datasets, thus learning to model a wide range of temporal patterns. Chronos demonstrates superior performance across 42 benchmark datasets, outperforming in-domain and zero-shot scenarios.

## Data and methods

### Datasets

This section details the dataset employed to evaluate the predictive performance of deep learning and foundation models, with real-world traffic data collected via a Split Cycle Offset Optimisation Technique (SCOOT) based Urban Traffic Control system^47^. The SCOOT uses a network of sensors to capture traffic flow data across the road network. The dataset includes traffic flows from the Glasgow City Council area over four consecutive years, from 1 October 2019 to 30 September 2023, which includes the COVID-19 pandemic period^9^. The SCOOT dataset contains 470 sensors that record traffic flows at 60-min intervals.

**Fig. 1:**
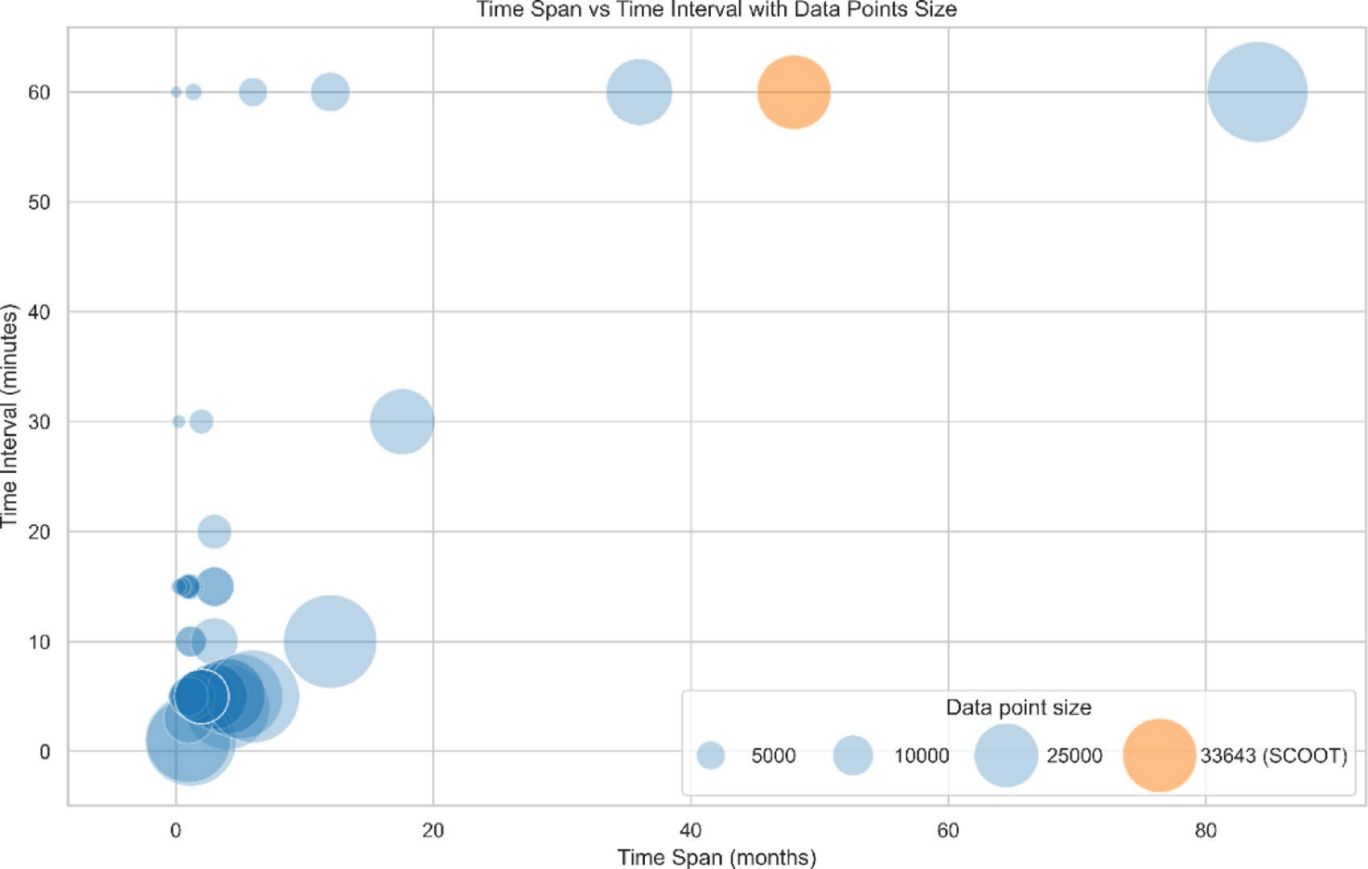
Time span, time interval, and data volume of traffic flow datasets.

**Fig. 2:**
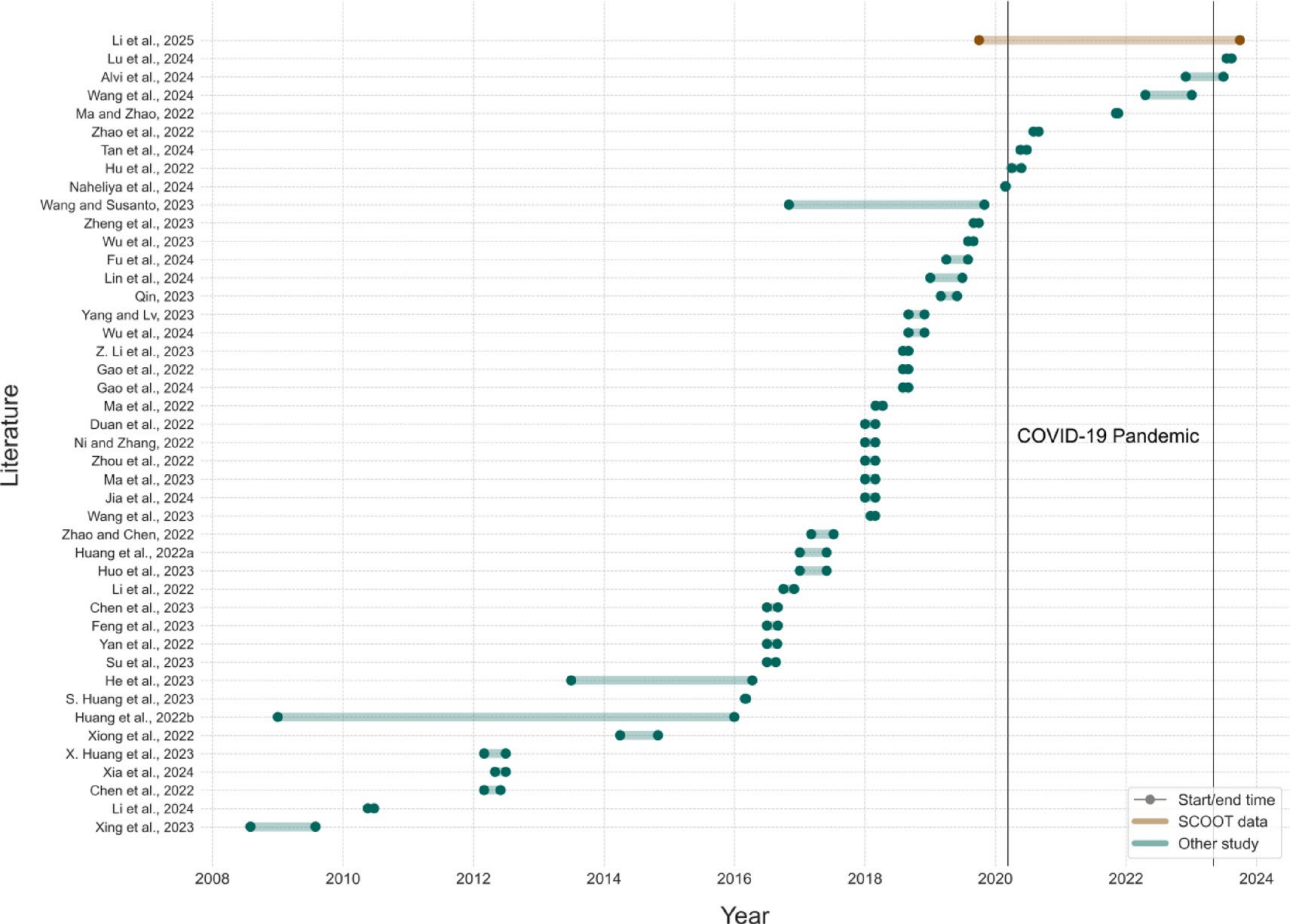
Time scope of traffic flow datasets.

Figures 1 and 2 compare the attributes of the SCOOT dataset with those of other traffic flow datasets used in recent traffic flow prediction research from 2022 to 2024^15,22,31,54,76–85^. Most datasets cover no more than one year during normal periods and use time intervals of less than 30 min^21,86–96^, while the SCOOT dataset covers longer than many previous studies. Although one study utilised a seven-year traffic datase^79^, which is longer than the SCOOT dataset, it only covers a period of stable traffic conditions. In contrast, the SCOOT dataset provides traffic flows before, during, and after COVID-19, providing valuable insights into the drastic changes in human mobility patterns in response to government mobility interventions during a period of significant disruption. Its long-term coverage enables models to learn long-term traffic trends, seasonal fluctuations, and emergency-related variations, thereby improving predictive performance. Additionally, the bubble size in Fig. 1 corresponds to the number of data points, and the SCOOT dataset contains a relatively large number of observations. This large volume of data enhances deep learning and foundation models by improving their ability to learn complex traffic patterns and reducing the risk of overfitting.

### Deep learning and foundation models

We select three widely used deep learning models for time series analysis, CNN^97^, LSTM^98^ and the transformer-based model Informe^18^, as well as two recently developed time series foundation models, Lag-Llama^46^ and Chronos^23^, to assess the performance of traffic flow prediction. The details of the models are outlined as follows:

#### Convolutional neural network (CNN)

CNN^97^ captures temporal patterns by applying convolutional filters to sliding windows of sequential data. This approach effectively detects local trends and short-term dependencies, enhancing prediction accuracy. In our CNN model, two one-dimensional convolutional layers are employed, with each utilising the ReLU activation function. The first layer specifies the input shape based on the sequence length and extracts initial local features from the traffic flow data. A subsequent convolutional layer further refines these features. A max-pooling layer then reduces the dimensionality of the resulting feature maps, preserving essential representations while reducing computational cost. Finally, the network is flattened and regularised with a dropout layer to prevent overfitting, followed by a dense layer that produces forecasts over 6 time steps.

#### Long short-term memory (LSTM)

LSTM^98^ effectively models long-term dependencies and sequential relationships in time series data via gated memory cells that retain relevant historical information while addressing vanishing gradient issues. Our LSTM model includes two hidden layers. The first LSTM layer, configured with the tanh activation function and set to return sequences, processes the input sequence to extract temporal features and passes the entire sequence to the subsequent layer. The second LSTM layer further refines these features using the tanh activation function. Each LSTM layer is followed by a dropout layer to reduce the risk of overfitting. Finally, a dense layer with six neurons is employed for multi-step prediction.

#### Informer

Informe^18^ is a transformer-based model specifically designed for long-sequence time series forecasting. Unlike standard transformers, which suffer from quadratic computational complexity with respect to sequence length, Informer introduces a probabilistic sparse self-attention mechanism that selects the most informative query–key pairs, significantly reducing memory usage and computational cost. In addition, Informer applies distilling operations between attention layers to progressively reduce sequence length while preserving salient temporal information. Informer incorporates a generative decoder structure that produces multi-step forecasts in a single forward step rather than step-by-step, significantly improving inference speed for multi-step prediction tasks. In this study, the Informer model contains two encoder layers and one decoder layer, with a batch size of 32.

#### Lag-Llama

Lag-Llama^46^ is a foundation model for univariate probabilistic time series prediction, built on a decoder-only transformer architecture, LLaMA^38^. Lag-Llama was released on 7 February 2024 and contained 2.45 million parameters. Lag-Llama tokenises input data by constructing lagged feature vectors using historical observations at predetermined lag intervals. These intervals include multiple standard frequencies such as quarterly, monthly, weekly, daily, hourly, and second-level frequencies. Each token also incorporates temporal covariates derived from date-time features such as hour-of-day, day-of-week, and month-of-year, enriching the representation and providing contextual information to the model. The input tokens, composed of lagged features and temporal covariates, are projected into a hidden representation and passed through a series of causally masked transformer decoder layers, employing RMSNorm and Rotary Positional Encoding (RoPE) at each attention layer. The final output from the transformer decoder is fed into a distribution head that predicts the parameters of a Student’s t-distribution (degrees of freedom, mean, and scale) for probabilistic forecasting. Detailed definitions of the lag structures and temporal covariates, including the specific lag intervals and feature construction process, are provided in the original Lag-Llama paper^46^.

Lag-Llama applies a robust scaling procedure using median and interquartile range (IQR) normalisation to handle numerical scale variations across different time series, significantly improving training stability and forecast accuracy. During training, Lag-Llama minimises the negative log-likelihood of the forecast distribution for future values. Lag-Llama is pre-trained on 27 datasets categorised into six domains: air quality, transportation, economics, nature, energy, and cloud operations. The pre-training corpus comprises 7,965 univariate series totaling about 352 million data tokens. This extensive and diverse corpus improves Lag-Llama’s ability to generalise and deliver strong zero-shot forecasting performance.

#### Chronos

Chronos^23^ is a pre-trained probabilistic forecasting framework designed specifically for time series, built on transformer-based language models. The core innovation of Chronos is its approach to treating time-series forecasting similarly to natural language modeling. It achieves this by tokenising continuous time series data into discrete tokens using a two-step approach: scaling and quantisation. Firstly, Chronos tokenises time series data by scaling each series individually using mean scaling, which normalises the data based on the mean of absolute historical values. Then, the scaled data are quantised into discrete bins, forming tokens from a fixed-size vocabulary. This vocabulary includes numerical bins and special tokens such as PAD (for padding sequences to equal lengths) and EOS (end-of-sequence). Detailed descriptions of the tokenisation and scaling procedures, including bin construction and scaling strategies, are provided in the original Chronos paper^23^.

The Chronos modelling framework applied in this study was released on 13 March 2024. Chronos primarily employs variants of the T5 family of transformer-based language models, ranging from smaller models with approximately 8 million parameters to larger models of up to 710 million^74^. These models are trained in 5 sizes, named Tiny (8 M), Mini (20 M), Small (46 M), Base (200 M) and Large (710 M), using a cross-entropy loss function, effectively framing regression as a classification task over discrete quantised bins. Chronos models provide probabilistic forecasts by autoregressively sampling from learned categorical distributions and subsequently mapping the sampled tokens back to continuous numerical values via dequantisation and inverse scaling. To enhance training, Chronos utilises data augmentation methods: TSMixup, which creates augmented series through convex combinations of existing series, and KernelSynth, which generates synthetic series using Gaussian processes. Chronos was pre-trained on 28 datasets, comprising publicly available datasets spanning transport, retail, energy, finance, healthcare, and climate science, complemented by synthetic datasets. The comprehensive benchmark evaluation involved 42 datasets to assess in-domain and zero-shot forecasting performance.

### Model implementations

To evaluate the model’s performance on usual traffic patterns and unusual traffic dynamics, we divide the SCOOT dataset into two subgroups: the entire dataset, including the pandemic period, and the post-COVID-19 dataset. Based on the Stringency Index, the entire dataset contains hourly traffic flow data from 1 October 2019 to 30 September 2023, while the post-COVID-19 dataset includes data from 3 June 2022^99^. Each subgroup is chronologically divided into training (60%), validation (20%), and testing (20%) sets, with a 60‑minute interval for both training and prediction. To assess the impact of context length on prediction accuracy, we train models with varying context lengths. Specifically, the context length is set to 24 × *n* hours, where *n* ranges from 1 to 21, limited by the available computational memory. These context lengths are used to predict traffic flow over the next 6 h, a common forecasting horizon in existing research^20,86,100,101^.

We conduct experiments with different hyperparameters for each context length and dataset to train deep learning models, selecting the best-performing configuration for comparison. Informer is trained using its default model configuration. For all models, the evaluation metrics are computed at each forecast step and averaged across the six prediction steps to obtain the reported results. For deep learning models that produce deterministic point predictions, the evaluation metrics are further averaged across 10 independent inference runs to reduce the randomness of individual training runs. For foundation models, Lag-Llama generates 100 samples from its predictive distribution for each forecast step, while Chronos produces predicted quantile values at 10 fixed probability levels (0.1, 0.2, …, 0.9). The evaluation metrics for foundation models are therefore computed from aggregated predictions. The Adam optimizer is used for training all deep learning models, with CNN and LSTM models trained for 100 epochs and the Informer model trained for 20 epochs. The best hyperparameter settings for CNN and LSTM are summarised in Tables 1 and 2, respectively, while the variance of evaluation metrics for each model is recorded in Table 3. Mean Squared Error (MSE) is used as the loss function during the model training^102^:

1 $$Loss=\frac{1}{n}\sum_{i=1}^{n}{({\widehat{y}}_{i}-{y}_{i})}^{2}$$

All models are implemented in Python 3.12.4 and executed on a 64-bit Ubuntu server with an Intel Xeon Gold 6334 8-Core Processor × 2 @ 3.60 GHz CPU, 125 GB of RAM, and an NVIDIA A100 GPU with 24 GB of memory. The deep learning models are developed using TensorFlow 2.17.0, while Informer and the foundation models are implemented using PyTorch 2.3.1.

### Evaluation metrics

The accuracy of traffic prediction models is typically evaluated using performance metrics that quantify their ability to forecast traffic conditions. In this research, we employ three widely recognised metrics: Root Mean Square Error (RMSE), Mean Absolute Error (MAE), and Mean Absolute Percentage Error (MAPE). RMSE and MAE assess absolute errors, while MAPE evaluates relative errors^103^. Lower values in all metrics indicate better prediction performance. The formulas are as follows:

2 $$RMSE= \sqrt{\frac{1}{n}\sum_{i=1}^{n}{({\widehat{y}}_{i}-{y}_{i})}^{2}}$$

3 $$MAE=\frac{1}{n}\sum_{i=1}^{n}|{\widehat{y}}_{i}-{y}_{i}|$$

4 $$MAPE=\frac{1}{n}\sum_{i=1}^{n}|\frac{{\widehat{y}}_{i}-{y}_{i}}{{y}_{i}}|\times 100$$

where ${y}_{i}$ and ${\widehat{y}}_{i}$ represent the ground truth and the predicted value for the $n$ th traffic flow sample. $n$ is the total number of the prediction samples.

## Results

### Model performance comparison

**Fig. 3:**
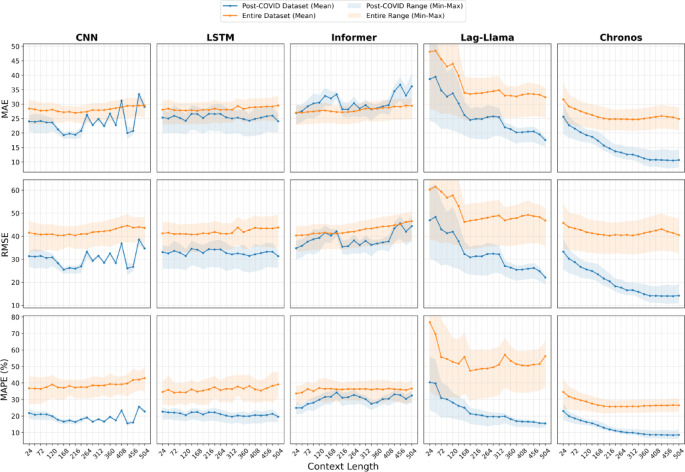
Comparison of the post-COVID-19 and entire dataset performance (MAE, RMSE, MAPE) of models across different input lengths.

This study compares the performance of traffic flow prediction models across the entire dataset and the post-COVID-19 dataset at different input lengths (context lengths) for both deep learning and foundation models (Fig. 3). The evaluation results clearly distinguish between model performance when trained on the post-COVID-19 dataset and when trained on the entire dataset. Across all models, evaluation metrics (MAE, MAPE and RMSE) are consistently lower for the post-COVID-19 dataset. This suggests that deep learning and foundation models perform better with stable traffic patterns. Specifically, the improvements in deep learning models are moderate, with slight decreases in RMSE and MAPE when evaluated on the post-COVID-19 dataset, suggesting that deep learning models may be less sensitive to different data patterns. In contrast, foundation models exhibit a noticeable performance gap across datasets. When predicted on post-COVID-19 data, the reduction in MAE, MAPE and RMSE is more evident than deep learning models, particularly for Lag-LLaMA, indicating improved model adaptability to stable traffic dynamics.

According to the context length, increasing it improves prediction performance for foundation models, whereas only limited improvements are observed for deep learning models. CNN, LSTM and Informer generally exhibit poorer performance as the context length increases. Although LSTM maintains a relatively stable trend, CNN and Informer show greater performance fluctuations. In particular, CNNs exhibit pronounced variability at longer context lengths, suggesting potential overfitting or inefficiencies in capturing long-term dependencies. Foundation models consistently reduce MAE, MAPE and RMSE as context length increases, although with slight fluctuations. Lag-LlaMA, in particular, demonstrates the most considerable improvement, reinforcing its ability to apply long historical sequences effectively. These findings highlight the superior capacity of foundation models to process and utilise long-term dependencies in time-series prediction.

Although foundation models generally benefit from longer context lengths, their performance declines when the context is short—often falling behind deep learning models. In particular, Lag-LLaMA consistently yields higher MAE and RMSE values than deep learning models across all context lengths when evaluated on the full dataset. This can be attributed to the zero-shot nature of these pre-trained models, which rely on broadly learned universal patterns from large-scale, high-quality data rather than task-specific training^11^. The entire dataset, which includes more heterogeneous and unusual traffic patterns, would make zero-shot prediction more demanding for these models. In contrast, the post-COVID-19 dataset exhibits more stable, universal traffic flow dynamics, enabling foundation models to utilise their extensive pre-training more effectively and outperform traditional deep learning models. Besides, the consistent performance improvements observed with longer context lengths demonstrate the importance of providing foundation models with sufficient historical information to enhance their zero-shot predictions in time series prediction.

### Training time and inference time analysis

**Fig. 4:**
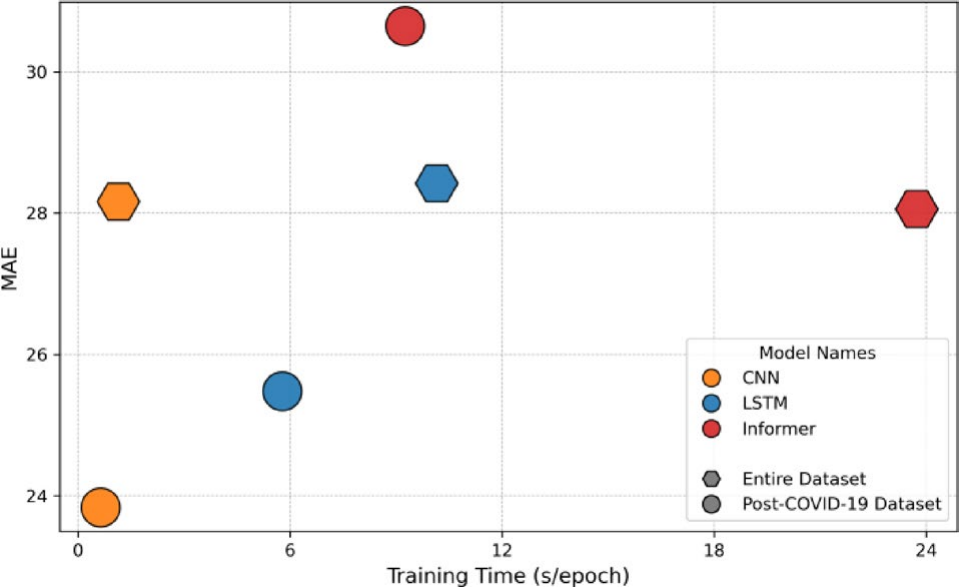
Average training time and MAE of CNN, LSTM and Informer across all context lengths (measured on NVIDIA A100 GPU).

Figure 4 illustrates the trade-off between training time and Mean Absolute Error (MAE) for deep learning models on the post-COVID-19 and entire datasets. For the post-COVID-19 dataset, CNN demonstrates the fastest training time at less than 1 s per epoch while achieving the best prediction performance. LSTM requires a moderately longer training time of around 6 s per epoch, but delivers poorer performance than CNN. Notably, Informer exhibits the longest training time, yet paradoxically achieves the poorest prediction performance. This indicates that with relatively stable traffic patterns, increased model complexity and training time do not necessarily lead to improved accuracy, with the most computationally intensive model showing the weakest performance. In contrast, the entire dataset exhibits a different pattern. Although the training time trend remains consistent across models, both LSTM and Informer show modest performance improvements over CNN, with all models achieving MAE values of around 28. This suggests that when dealing with more complex and irregular traffic patterns, the sophisticated temporal modelling capabilities of LSTM and Informer provide limited but observable benefits, though these improvements come at the cost of substantially increased computational demands.

**Fig. 5:**
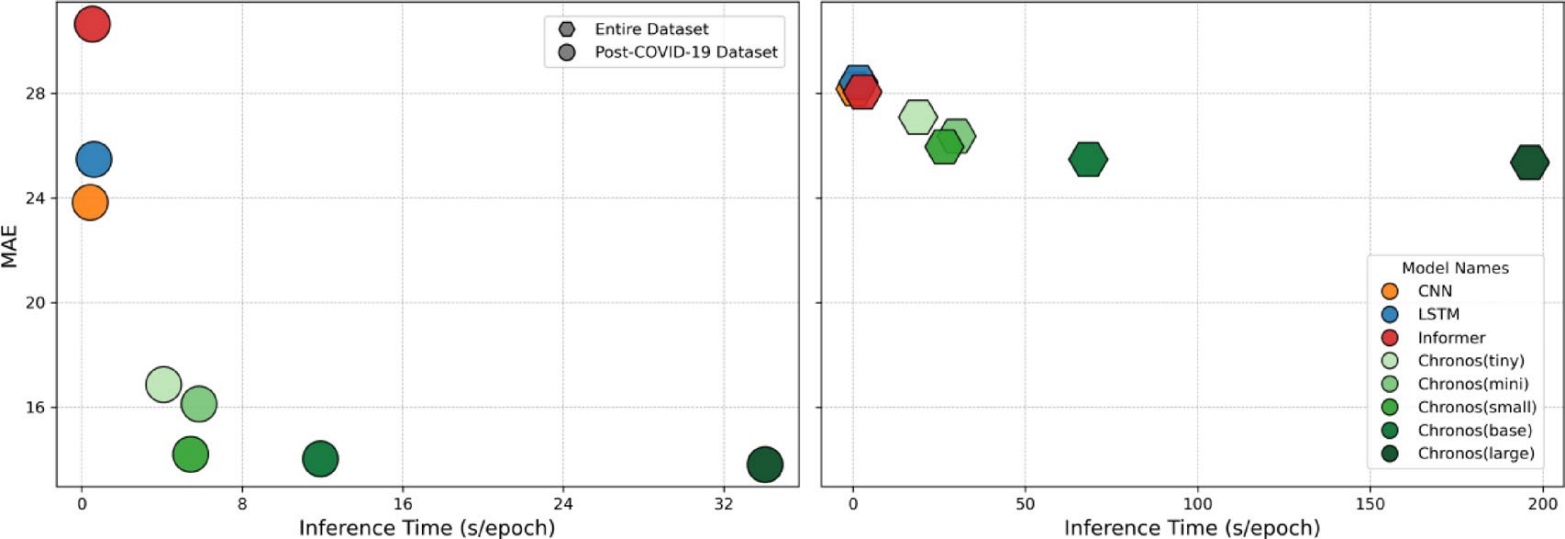
Average inference time and MAE of models across all context lengths (measured on NVIDIA A100 GPU).

We also compare the inference time for all the models, including deep learning and foundation models (Fig. 5). It is important to note that Lag-Llama is intentionally omitted from this figure due to its exceptionally high inference times^5^. It requires approximately 132 s per epoch on the post-COVID-19 dataset and 875 s per epoch on the entire dataset, substantially longer than the inference times observed for the other models. For both post-COVID-19 and entire datasets, deep learning models exhibit relatively short inference times but moderately higher MAEs, while the Chronos models show a broader range of inference times that generally increase with model size. Unlike the training time comparison, where Informer required significantly longer training times than CNN and LSTM, all three deep learning models show similar inference durations. This is because they all produce predictions through a single forward procedure during inference. While Informer’s training is computationally intensive due to its complex self-attention mechanisms, its generative-style decoder enables efficient multi-step forecasts comparable to those of CNNs and LSTMs.

For Chronos models, larger configurations tend to achieve lower MAEs at the cost of progressively longer inference durations. This trade-off stems from Chronos’s autoregressive forecasting method, where future values are generated sequentially by sampling from learned probabilistic distributions^23^. This sequential generation process, combined with increased parameter counts in larger models, leads to substantially extended inference times. A key observation is that Chronos (Small) achieves shorter inference times and better performance than Chronos (Mini), indicating that this configuration effectively balances computational efficiency and predictive accuracy. However, beyond Chronos (Small), increasing model size brings only marginal performance improvements while substantially increasing inference time, suggesting diminishing returns for larger Chronos variants in traffic flow prediction tasks.

### Model size analysis

**Fig. 6:**
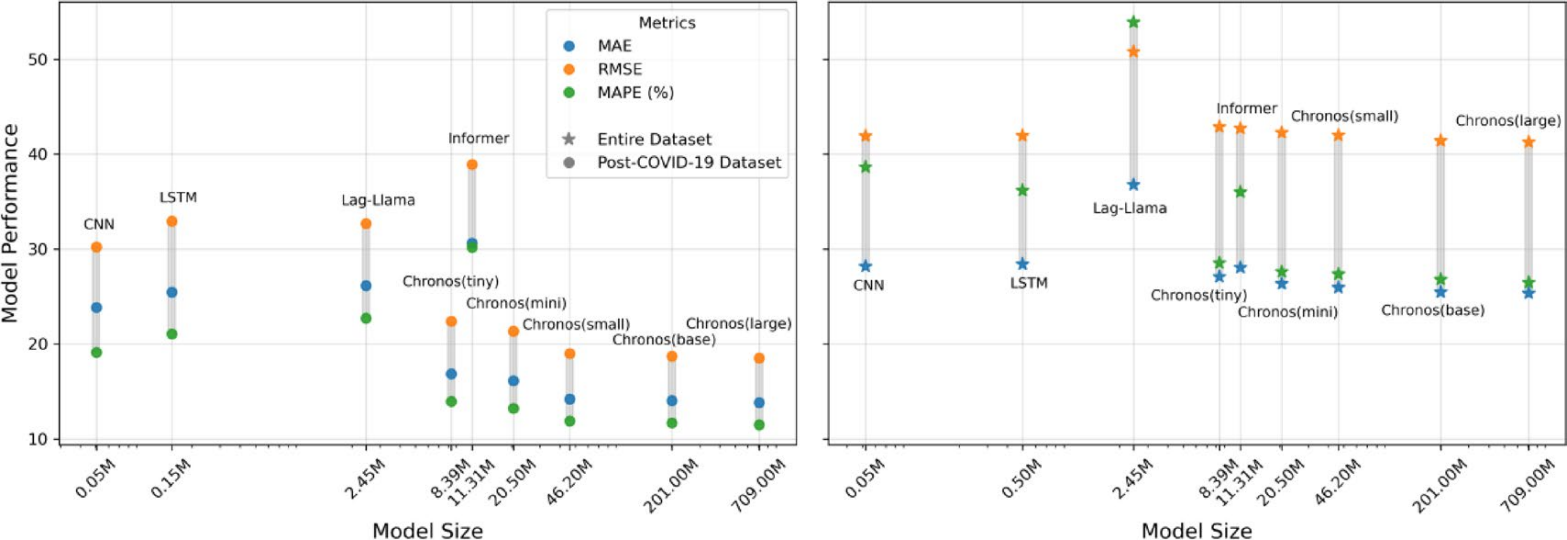
Comparison of the post-COVID-19 and entire dataset performance of models across different model sizes, averaged across all context lengths.

Figure 6 compares the average performance across different context lengths as a function of model size, measured by the number of parameters. Specifically, the number of parameters in deep learning models depends on their architecture, hyperparameters, and context length, whereas foundation models maintain a fixed parameter count. From Fig. 6, it is clear that deep learning models and foundation models demonstrate different performance trends as their sizes change. For foundation models, increasing the number of parameters generally improves prediction accuracy on both the post-COVID-19 dataset and the entire dataset, suggesting that additional parameters help learn complex traffic patterns. In contrast, deep learning models show a negative correlation between model size and prediction accuracy on the post-COVID-19 dataset. Specifically, CNN achieves the best performance, followed by LSTM, while Informer performs the worst despite having substantially more parameters than both CNN and LSTM. Across the entire dataset, all three deep learning models converge to similar performance levels regardless of model size.

## Discussion

In this study, we compare the performance of deep learning models and cutting-edge foundation models for traffic flow prediction. The deep learning models, CNN, LSTM and Informer, are trained on the SCOOT dataset ourselves, while Lag-Llama and Chronos are pre-trained time series foundation models, which can be applied for zero-shot prediction. We have found that foundation models with longer context lengths and larger model sizes tend to achieve higher prediction accuracy, while deep learning models show limited improvement. This suggests that deep learning models may overfit or struggle to capture long-term dependencies. In contrast, foundation models demonstrate a superior ability to process and utilise long-term dependencies in time-series prediction. However, there is a trade-off between model performance and inference time, as increasing context length and model size require more substantial computational resources. Moreover, although foundation models are more sensitive to traffic patterns, they outperform deep learning models in both usual and unusual traffic conditions, with Chronos demonstrating particularly strong performance.

**Fig. 7:**
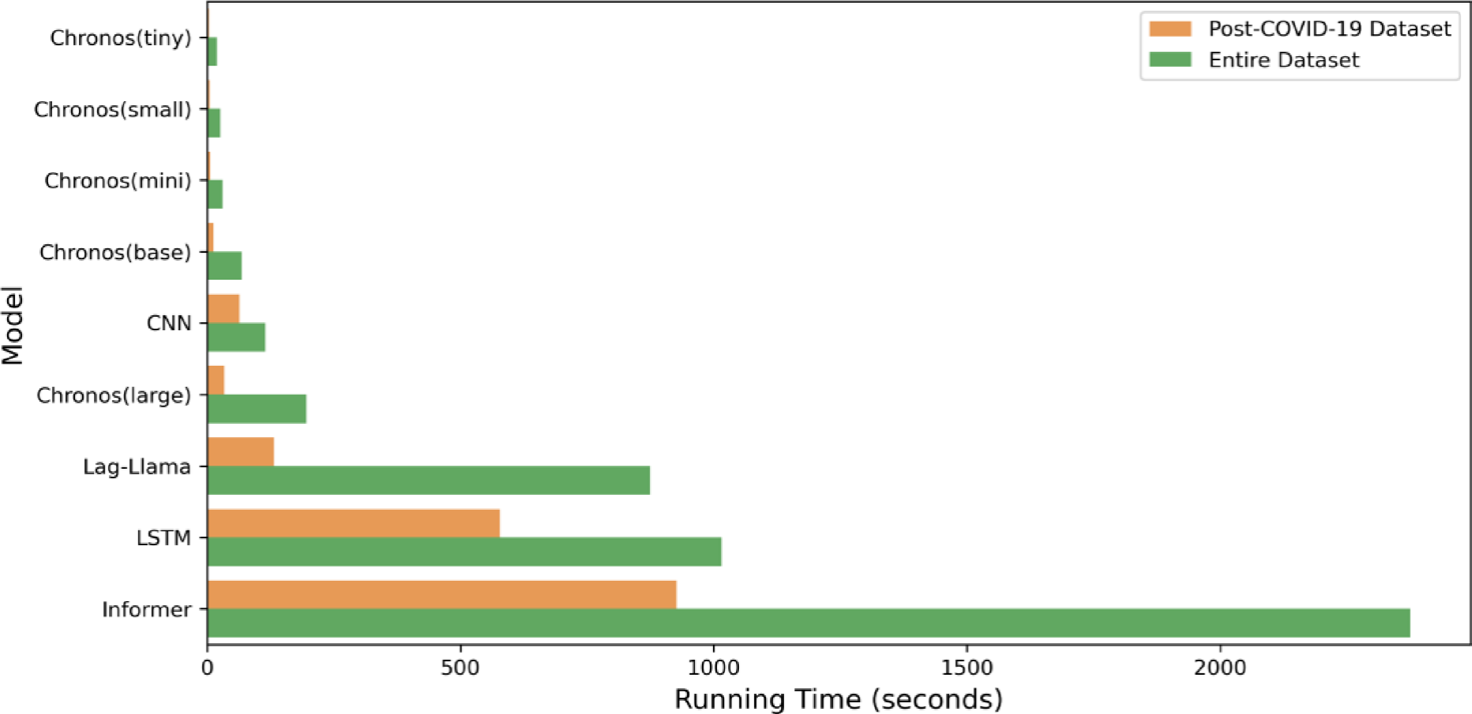
Comparison of the cumulative running time of models, averaged across all context lengths (measured on NVIDIA A100 GPU).

In our experiments, we evaluate training and inference times separately to provide a clear understanding of the computational demands of each stage. However, for a comprehensive comparison between deep learning models and foundation models, we calculate the total running time by combining the training and inference durations for the deep learning models. For foundation models that are not trained in this study, the running time is limited to inference time only. Specifically, we calculate the cumulative running time over 100 training epochs for deep learning models and one prediction epoch for all models. As shown in Fig. 7, Chronos’s running times are significantly lower than those of other models, particularly on the small dataset. While the running time of CNN is shorter than that of Lag-Llama in this case, it overlooks hyperparameter tuning, which is essential in the deep learning training process for each prediction task. The tuning process involves testing dozens of hyperparameter combinations^104,105^, with each test requiring a time equivalent to the running time observed here (since the inference times of deep learning models are too short to be considered). This leads to a practical running time multiple times greater than the running time here. As a result, the runtime of foundation models is shorter than that of deep learning models. Besides, deep learning models require separate training for each prediction task. In contrast, foundation models can be directly applied to different prediction tasks across various datasets with varying context lengths. This significantly simplifies model deployment and streamlines forecasting pipelines, eliminating the need for task-specific training.

**Fig. 8:**
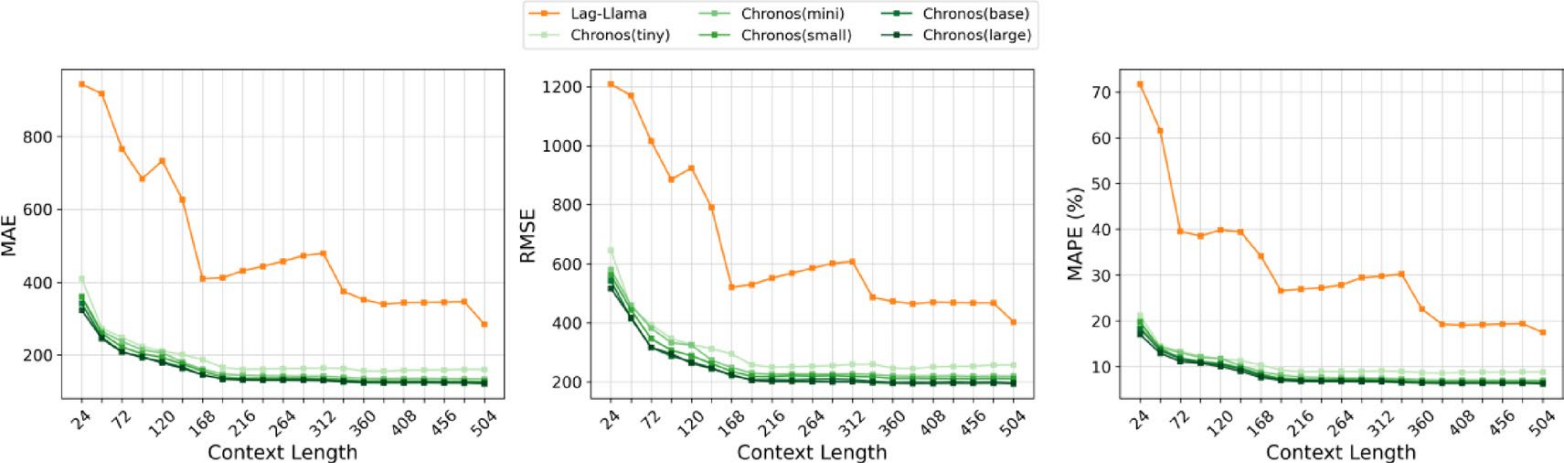
Comparison of model performance across different context lengths.

A key limitation of foundation models is the performance gap between different models. Our findings indicate that Lag-Llama shows only limited improvements in prediction accuracy compared to deep learning models, while Chronos consistently demonstrates strong performance across various context lengths. Lag-Llama also shows lower stability than Chronos, with a sharper decrease in accuracy at longer prediction steps. To further illustrate this performance gap, we used another publicly available, widely used traffic flow dataset collected by the Caltrans Performance Measurement System (PeMS) in California, USA, from 1 January to 31 December, 2018. As shown in Fig. 8, the results are consistent with those from the SCOOT dataset in our research, with Chronos significantly outperforming Lag-Llama across all context lengths. Since both Chronos and Lag-Llama are pre-trained on diverse publicly available datasets, the observed performance difference may stem from their respective training data. Comparing their datasets, we find that Chronos is trained on seven datasets, while Lag-Llama uses only three. Additionally, Chronos incorporates synthetic data generated by Gaussian processes to enhance its training. The training data for Chronos spans 2009–2022, including the 2020–2021 pandemic period, enabling the model to learn mobility patterns amid pandemic-related disruptions. Lag-Llama’s training data is limited to 2009 and 2014–2016 and therefore does not capture pandemic-related mobility changes. This suggests that training foundation models on a larger, more temporally diverse corpus of time-series data improves zero-shot performance. Moreover, model size plays a crucial role in performance. Chronos offers models ranging from 8 M (Tiny) to 710 M (Large) parameters, which are significantly larger than Lag-Llama’s 2.45 M parameters and are likely contributing to its superior predictive accuracy. However, it is worth noting that while Chronos shows consistently better performance with increasing model size, the improvements from the Small to the Large variants are modest, suggesting a degree of model saturation for this prediction task. The univariate nature of the input and the limited complexity of short-horizon traffic flow prediction may constrain the benefit of adding substantially more parameters. Future work could explore whether multivariate prediction tasks enable larger foundation model variants to realise greater performance increases.

In summary, this research highlights that foundation models can achieve excellent zero-shot performance in traffic flow prediction under both normal conditions and disruptive events. Unlike deep learning models, which require extensive task-specific training and domain expertise, foundation models can be directly applied to datasets with different data sizes, traffic dynamics, and context lengths. Besides, while deep learning models require large-scale historical data for training and validation, foundation models can make accurate zero-shot predictions with only a small subset of contextual data. Those advantages can address critical limitations of deep learning methods, such as time-consuming training processes, overfitting due to task-specific model training, and limited generalisation capabilities. Additionally, they can support the practical deployment of traffic prediction models across diverse, dynamically changing urban environments. However, choosing an appropriate foundation model is crucial, as performance depends on factors such as the diversity of the training data, the time coverage, and the model size. A well-trained foundation model with a comprehensive dataset is more likely to achieve superior zero-shot performance, making it a practical and efficient choice for real-world traffic prediction applications.

Using foundation models for traffic flow prediction and other time series analysis tasks in urban settings presents both opportunities and challenges. The development of foundation models has been one of the fastest-growing areas over the past two and a half years, since OpenAI released the first version of ChatGPT 3.5 in November 2022. As foundation models continue to evolve in both capability and efficiency, the prediction accuracy of time-series foundation models is expected to improve accordingly. Accordingly, the results presented in this study represent a baseline, reflecting the performance of the foundation models available at the time of experimentation in a zero-shot setting. This study focuses on non-spatial time-series models in a single-sensor forecasting setting because current foundation models do not natively incorporate spatial information; therefore, our findings should be interpreted within this specific experimental scope rather than as a replacement for spatial–temporal models, which remain essential for capturing network-level traffic dynamics. Future research may extend these experiments with more recent models and adaptation strategies such as fine-tuning or few-shot learning, and explore spatial–temporal architectures to further improve prediction accuracy and better capture the underlying dynamics of urban traffic systems. As we observed in this research, although foundation models exhibit superior performance in this research, with faster inference and higher accuracy, they still have several limitations. Firstly, the training process of foundation models is typically very expensive and time-consuming, making it less accessible for academic institutions or small research groups to extend or revise pre-trained models.

Furthermore, the efficiency of foundation models heavily relies on the underlying pre-trained architectures, and the most advanced foundation models are often closed-source and developed by leading generative AI companies. As a result, most researchers can only rely on “less advanced” or “older generation” publicly available foundation models to design fine-tuned models for time series prediction. In the future, a better collaboration between AI companies and academia is necessary to enable further customised model development. Lastly, the limited scope and diversity of training data^23^ for time-series foundation models lead to performance disparities and prediction biases across foundation models. Future work can focus on building and maintaining large-scale, diverse traffic datasets to improve model training and predictive accuracy across various scenarios. The model can also be further evaluated in regions with limited data, such as those in the Global South, to assess the effectiveness of using foundation models trained on datasets from Global North countries in different geographical contexts. Governments can encourage collaborative data-sharing initiatives between public and private sectors to expand the availability of high-quality traffic data for model development.

## Data Availability

The data used in this paper is publicly available. Full details about the data acquisition can be found in the documentation available at the GitHub repository: (https://github.com/YueLi-0816/trafficFlowPrediction) .

## Appendix 1: Hyperparameter tuning

Tables 1 and 2 present the optimal hyperparameters for the CNN and LSTM, respectively, selected based on the lowest training loss observed during training. These hyperparameters are fine-tuned to achieve optimal model performance across different context lengths and datasets.

**Table 1:** Hyperparameters of CNN.

Context Length	Model Hyper-parameter Dataset	CNN								
	Filters		Kernel Size		Dropout Rate		Learning Rate		Batch Size	
	Post-COVID	Entire	Post-COVID	Entire	Post-COVID	Entire	Post-COVID	Entire	Post-COVID	Entire
24	128	128	3	3	0.3	0.3	0.0005	0.0001	64	64
48	64	128	3	3	0.0	0.3	0.0005	0.0001	128	128
72	64	128	5	5	0.3	0.1	0.001	0.0001	64	64
96	64	64	3	5	0.0	0.3	0.0005	0.0001	128	32
120	64	64	3	5	0.1	0.2	0.0005	0.0005	128	128
144	32	64	2	5	0.3	0.3	0.0005	0.0001	32	128
168	32	64	2	5	0.1	0.3	0.0005	0.0005	64	64
192	128	32	3	5	0.2	0.3	0.0001	0.001	32	128
216	64	64	3	5	0.2	0.3	0.0001	0.0001	32	64
240	32	32	5	3	0.0	0.3	0.0001	0.0001	32	32
264	128	64	3	3	0.3	0.3	0.0005	0.0001	32	128
288	32	128	3	2	0.3	0.3	0.0001	0.001	32	128
312	32	32	2	3	0.1	0.3	0.0005	0.0005	128	128
336	64	32	2	3	0.2	0.3	0.0001	0.0001	32	128
360	32	32	2	2	0.3	0.2	0.0001	0.0001	64	32
384	32	32	2	3	0.1	0.3	0.0001	0.0001	128	64
408	32	32	2	3	0.1	0.3	0.001	0.0001	128	64
432	32	32	3	2	0.0	0.2	0.0001	0.0001	32	64
456	32	32	3	2	0.0	0.3	0.0001	0.0001	64	128
480	64	32	2	3	0.2	0.3	0.0001	0.0001	32	128
504	32	32	2	2	0.1	0.2	0.0001	0.0001	64	64

**Table 2:** Hyperparameters of LSTM.

Context Length	Model	LSTM							
	Hyper-parameter	Hidden Units		Dropout Rate		Learning Rate		Batch Size	
	Dataset	Post-COVID	Entire	Post-COVID	Entire	Post-COVID	Entire	Post-COVID	Entire
24		32	64	0.2	0.2	0.001	0.001	32	32
48		128	256	0.2	0.2	0.01	0.01	64	32
72		256	256	0.0	0.1	0.001	0.001	32	128
96		64	256	0.2	0.4	0.01	0.001	32	32
120		32	128	0.2	0.1	0.001	0.001	32	64
144		128	256	0.3	0.2	0.01	0.001	32	64
168		32	256	0.0	0.3	0.0005	0.001	32	64
192		64	256	0.2	0.4	0.01	0.001	128	32
216		128	256	0.1	0.4	0.01	0.001	32	32
240		128	64	0.1	0.4	0.01	0.005	32	64
264		128	256	0.3	0.4	0.01	0.001	32	32
288		128	64	0.0	0.3	0.01	0.001	32	64
312		64	32	0.2	0.2	0.01	0.001	64	32
336		128	256	0.3	0.1	0.01	0.01	32	64
360		128	32	0.2	0.1	0.001	0.001	32	32
384		64	256	0.2	0.3	0.01	0.001	32	64
408		128	256	0.2	0.4	0.01	0.001	32	64
432		64	128	0.3	0.4	0.01	0.001	32	64
456		64	128	0.3	0.0	0.01	0.001	32	32
480		32	256	0.4	0.1	0.01	0.001	32	64
504		128	128	0.4	0.0	0.01	0.001	32	32

## Variance analysis of evaluation metrics

Table 3 presents the variance of evaluation metrics (MAE, RMSE, and MAPE) for all models. For deep learning models (CNN, LSTM, and Informer), the variance is computed across 10 independent inference runs to assess the stability and reproducibility of model predictions. For foundation models (Chronos and Lag-Llama), which produce deterministic outputs for a given input, variance across runs is not applicable. Instead, for all models, we report the variance of evaluation metrics across the six forecast steps, which reflects how prediction performance varies with increasing prediction horizons. Lower variance indicates more consistent performance, while higher variance suggests that model accuracy degrades or fluctuates. In particular, Lag-Llama exhibits relatively high variance, indicating that its prediction accuracy decreases markedly as the forecast horizon increases, while other models show relatively low and similar variance, with more stable performance across different prediction steps.

**Table 3:** Variance of evaluation metrics for each model.

Model	Dataset	Post-COVID			Entire		
	Metrics (Variance across 10 runs)	MAE	RMSE	MAPE	MAE	RMSE	MAPE
CNN		5.424	5.156	3.074	0.083	0.039	2.722
LSTM		4.796	6.401	3.238	0.311	0.732	4.795
Informer		9.641	11.948	8.718	0.285	0.236	8.297
CNN		1.876	2.778	1.402	5.997	16.718	24.111
LSTM		11.191	16.746	7.581	8.020	21.333	29.672
Informer		5.950	8.721	6.945	6.225	18.310	19.957
Lag-Llama		29.133	39.589	28.083	58.478	88.254	216.793
Chronos (Tiny)		1.624	3.104	1.200	7.087	23.276	9.922
Chronos (Mini)		1.339	2.623	0.947	6.798	24.966	8.710
Chronos (Small)		1.451	2.741	1.104	6.690	24.741	8.924
Chronos (Base)		1.573	3.001	1.219	6.292	23.642	8.725
Chronos (Large)		1.445	2.727	1.204	6.148	23.358	8.047

## References

[1] Yin, X. et al. Deep learning on traffic prediction: Methods, analysis, and future directions. *IEEE Trans. Intell. Transp. Syst.* **23**, 4927–4943 (2022).

[2] Liu, Y., Rasouli, S., Wong, M., Feng, T. & Huang, T. RT-GCN: Gaussian-based spatiotemporal graph convolutional network for robust traffic prediction. *Inf. Fusion.* **102**, 102078 (2024).

[3] Chen, H. & Rakha, H. A. Real-time travel time prediction using particle filtering with a non-explicit state-transition model. *Transp. Res. C. Emerg. Technol.* **43**, 112–126 (2014).

[4] Guo, G. & Yuan, W. Short-term traffic speed forecasting based on graph attention temporal convolutional networks. *Neurocomputing* **410**, 387–393 (2020).

[5] Liu, C. *et al.* Spatial-temporal large language model for traffic prediction. Preprint at 10.48550/arXiv.2401.10134 (2024).

[6] Kim, Y., Tak, H., Kim, S. & Yeo, H. A hybrid approach of traffic simulation and machine learning techniques for enhancing real-time traffic prediction. *Transp. Res. C. Emerg. Technol.* **160**, 104490 (2024).

[7] Chen, J. et al. Traffic flow matrix-based graph neural network with attention mechanism for traffic flow prediction. *Inf. Fusion.* **104**, 102146 (2024).

[8] Fan, J. et al. RGDAN: A random graph diffusion attention network for traffic prediction. *Neural Netw.* **172**, 106093 (2024). 38228022 10.1016/j.neunet.2023.106093

[9] Li, Y., Zhao, Q. & Wang, M. Understanding urban traffic flows in response to COVID-19 pandemic with emerging urban big data in Glasgow. *Cities* **154**, 105381 (2024).

[10] Kalašová, A. & Stacho, M. Smooth traffic flow as one of the most important factors for safety increase in road transport. *Transport*10.3846/16484142.2006.9638037 (2006).

[11] Ren, Y. *et al.* TPLLM: A Traffic Prediction Framework Based on Pretrained Large Language Models. Preprint at 10.48550/arXiv.2403.02221 (2024).

[12] Sattarzadeh, A. R., Kutadinata, R. J., Pathirana, P. N. & Huynh, V. T. A novel hybrid deep learning model with ARIMA Conv-LSTM networks and shuffle attention layer for short-term traffic flow prediction. *Transportmet. A Transp. Sci.* **21**(1), 2236724 (2025).

[13] Zhang, Y., Tang, S. & Yu, G. An interpretable hybrid predictive model of COVID-19 cases using autoregressive model and LSTM. *Sci. Rep.* **13**, 6708 (2023). 37185289 10.1038/s41598-023-33685-z PMC10126574

[14] Wang, Y., Jia, R., Dai, F. & Ye, Y. Traffic flow prediction method based on seasonal characteristics and SARIMA-NAR model. *Appl. Sci.* **12**, 2190 (2022).

[15] Kashyap, A. A. et al. Traffic flow prediction models—A review of deep learning techniques. *Cogent Eng.*10.1080/23311916.2021.2010510 (2022).

[16] Li, Y., Chai, S., Ma, Z. & Wang, G. A hybrid deep learning framework for long-term traffic flow prediction. *IEEE. Access* **9**, 11264–11271 (2021).

[17] Méndez, M., Merayo, M. G. & Núñez, M. Long-term traffic flow forecasting using a hybrid CNN-BiLSTM model. *Eng. Appl. Artif. Intell.* **121**, 106041 (2023).

[18] Zhou, H*. et al.* Informer: Beyond efficient transformer for long sequence time-series forecasting. Preprint at 10.48550/arXiv.2012.07436 (2021).

[19] Li, Y. et al. Modeling temporal patterns with dilated convolutions for time-series forecasting. *ACM. Trans. Knowl. Discov. Data* **16**, 14:1-14:22 (2021).

[20] Wu, D., Peng, K., Wang, S. & Leung, V. C. M. Spatial-temporal graph attention gated recurrent transformer network for traffic flow forecasting. *IEEE. Internet Things J.* **11**, 14267–14281 (2024).

[21] Xia, Z., Zhang, Y., Yang, J. & Xie, L. Dynamic spatial-temporal graph convolutional recurrent networks for traffic flow forecasting. *Expert Syst. Appl.*10.1016/j.eswa.2023.122381 (2024).

[22] Zhao, Y. et al. Dual flow fusion graph convolutional network for traffic flow prediction. *Int. J. Mach. Learn. Cybern.* **15**, 3425–3437 (2024).

[23] Ansari, A. F. *et al.* Chronos: Learning the language of time series. Preprint at 10.48550/arXiv.2403.07815 (2024).

[24] Parr, S., Wolshon, B., Renne, J., Murray-Tuite, P. & Kim, K. Traffic impacts of the COVID-19 pandemic: Statewide analysis of social separation and activity restriction. *Nat. Hazards Rev.* **21**, 04020025 (2020).

[25] Warren, M. S. & Skillman, S. W. Mobility changes in response to COVID-19. Preprint at 10.48550/arXiv.2003.14228 (2020).

[26] Borkowski, P., Jażdżewska-Gutta, M. & Szmelter-Jarosz, A. Lockdowned: Everyday mobility changes in response to COVID-19. *J. Transp. Geogr.* **90**, 102906 (2021). 35721765 10.1016/j.jtrangeo.2020.102906 PMC9188832

[27] Nouvellet, P. et al. Reduction in mobility and COVID-19 transmission. *Nat. Commun.* **12**, 1090 (2021). 33597546 10.1038/s41467-021-21358-2 PMC7889876

[28] Patra, S. S., Chilukuri, B. R. & Vanajakshi, L. Analysis of road traffic pattern changes due to activity restrictions during COVID-19 pandemic in Chennai. *Transp. Lett.* **13**, 473–481 (2021).

[29] Ebrahim Shaik, M. & Ahmed, S. An overview of the impact of COVID-19 on road traffic safety and travel behavior. *Transp. Eng.* **9**, 100119 (2022).

[30] Hu, Y. *et al.* Impacts of Covid-19 mode shift on road traffic. Preprint at 10.48550/arXiv.2005.01610 (2023).

[31] Ma, C., Dai, G. & Zhou, J. Short-term traffic flow prediction for urban road sections based on time series analysis and LSTM_BILSTM method. *IEEE. Trans. Intell. Transp. Syst.* **23**, 5615–5624 (2022).

[32] Ghanim, M. S., Muley, D. & Kharbeche, M. ANN-based traffic volume prediction models in response to COVID-19 imposed measures. *Sustain. Cities Soc.* **81**, 103830 (2022). 35291578 10.1016/j.scs.2022.103830 PMC8906893

[33] Liapis, S. et al. A methodology using classification for traffic prediction: Featuring the impact of COVID-19. *Integr. Comput. Aided Eng.* **28**, 417–435 (2021).

[34] Li, H. et al. Traffic flow forecasting in the COVID-19: A deep spatial-temporal model based on discrete wavelet transformation. *ACM Trans. Knowl. Discov. Data* **17**, 1–28 (2023).

[35] Li, Z. et al. OpenCity: Open Spatio-temporal foundation models for traffic prediction. *ACM Trans. Intell. Syst. Technol.*10.1145/3773912 (2025).40575765

[36] Brown, T. *et al.* Language Models are Few-Shot Learners. in *Advances in Neural Information Processing Systems* vol. 33 1877–1901 (Curran Associates, Inc., 2020).

[37] Chung, H. W. *et al.**Scaling Instruction-Finetuned Language Models*. Preprint at 10.48550/arXiv.2210.11416 (2022).

[38] Touvron, H. *et al.**LLaMA: Open and Efficient Foundation Language Models*. Preprint at 10.48550/arXiv.2302.13971 (2023).

[39] Mirchandani, S. *et al.**Large Language Models as General Pattern Machines*. Preprint at 10.48550/arXiv.2307.04721 (2023).

[40] Gruver, N., Finzi, M., Qiu, S. & Wilson, A. G. *Large Language Models Are Zero-Shot Time Series Forecasters*. Preprint at 10.48550/arXiv.2310.07820 (2024).

[41] Liu, H., Zhao, Z., Wang, J., Kamarthi, H. & Prakash, B. A. *LSTPrompt: Large Language Models as Zero-Shot Time Series Forecasters by Long-Short-Term Prompting*. Preprint at 10.48550/arXiv.2402.16132 (2024).

[42] OpenAI *et al.**GPT-4 Technical Report*. Preprint at 10.48550/arXiv.2303.08774 (2024).

[43] Radford, A., Narasimhan, K., Salimans, T. & Sutskever, I. *Improving Language Understanding by Generative Pre-Training*. (2018).

[44] Radford, A. *et al.**Language Models are Unsupervised Multitask Learners*. (2019).

[45] Xue, H. & Salim, F. D. *PromptCast: A New Prompt-based Learning Paradigm for Time Series Forecasting*. Preprint at 10.48550/arXiv.2210.08964 (2023).

[46] Rasul, K. *et al.**Lag-Llama: Towards Foundation Models for Probabilistic Time Series Forecasting*. Preprint at 10.48550/arXiv.2310.08278 (2024).

[47] Li, Y., Zhao, Q. & Wang, M. High-resolution traffic flow data from the urban traffic control system in Glasgow.. *Sci. Data* **12**, 253 (2025). 39939620 10.1038/s41597-025-04494-y PMC11821839

[48] Park, J. *et al.* Real time vehicle speed prediction using a Neural Network Traffic Model. in *The 2011 International Joint Conference on Neural Networks* 2991–2996 (2011). 10.1109/IJCNN.2011.6033614.

[49] Jia, Y., Wu, J. & Du, Y. Traffic speed prediction using deep learning method. In *2016 IEEE 19th International Conference on Intelligent Transportation Systems (ITSC)* 1217–1222 (2016). 10.1109/ITSC.2016.7795712.

[50] Jia, Y., Wu, J., Ben-Akiva, M., Seshadri, R. & Du, Y. Rainfall-integrated traffic speed prediction using deep learning method. *IET Intell. Transp. Syst.* **11**, 531–536 (2017).

[51] Akhtar, M. & Moridpour, S. A review of traffic congestion prediction using artificial intelligence. *J. Adv. Transp.* **2021**, 8878011 (2021).

[52] Chen, C., Liu, Z., Wan, S., Luan, J. & Pei, Q. Traffic flow prediction based on deep learning in Internet of Vehicles.. *IEEE Trans. Intell. Transp. Syst.* **22**, 3776–3789 (2021).

[53] Aljebreen, M. et al. Enhancing traffic flow prediction in intelligent cyber-physical systems: A novel Bi-LSTM-based approach with Kalman filter integration. *IEEE Trans. Consum. Electron.* **70**, 1889–1902 (2024).

[54] Alvi, M., Minerva, R., Rajapaksha, P., Crespi, N. & Alvi, U. Traffic flow prediction in sensor-limited areas through synthetic sensing and data fusion. *IEEE Sens. Lett.*10.1109/LSENS.2024.3379311 (2024).

[55] Van Der Voort, M., Dougherty, M. & Watson, S. Combining Kohonen maps with ARIMA time series models to forecast traffic flow.. *Transp. Res. C Emerg. Technol.* **4**, 307–318 (1996).

[56] Okutani, I. & Stephanedes, Y. J. Dynamic prediction of traffic volume through Kalman filtering theory. *Transp. Res. B Methodol.* **18**, 1–11 (1984).

[57] Leshem, G. & Ritov, Y. Traffic Flow Prediction using Adaboost Algorithm with Random Forests as a Weak Learner. *Int. J. Electric. Comput. Eng.* **21**, (2007).

[58] Tang, J. et al. Traffic flow prediction based on combination of support vector machine and data denoising schemes. *Phys. A Stat. Mech. Appl.* **534**, 120642 (2019).

[59] Yang, S. & Qian, S. Understanding and predicting travel time with spatio-temporal features of network traffic flow, weather and incidents. *IEEE Intell. Transp. Syst. Mag.* **11**, 12–28 (2019).

[60] Tian, Y. & Pan, L. Predicting Short-Term Traffic Flow by Long Short-Term Memory Recurrent Neural Network. in *2015 IEEE International Conference on Smart City/SocialCom/SustainCom (SmartCity)* 153–158 (2015). 10.1109/SmartCity.2015.63.

[61] Zhu, H. et al. A novel traffic flow forecasting method based on RNN-GCN and BRB. *J. Adv. Transp.* **2020**, 7586154 (2020).

[62] Lu, S., Zhang, Q., Chen, G. & Seng, D. A combined method for short-term traffic flow prediction based on recurrent neural network. *Alex. Eng. J.* **60**, 87–94 (2021).

[63] Xiao, Y. & Yin, Y. Hybrid LSTM neural network for short-term traffic flow prediction. *Information*10.3390/info10030105 (2019).

[64] Wang, S., Zhao, J., Shao, C., Dong, C. D. & Yin, C. Truck traffic flow prediction based on LSTM and GRU methods with sampled GPS data. *IEEE Access* **8**, 208158–208169 (2020).

[65] Xiong, L., Ding, W., Huang, X. & Huang, W. CLSTAN: ConvLSTM-Based spatiotemporal attention network for traffic flow forecasting. *Math. Probl. Eng.*10.1155/2022/1604727 (2022).

[66] Wang, J.-D. & Susanto, C. O. N. Traffic flow prediction with heterogenous data using a hybrid CNN-LSTM model. *CMC-Comput. Mater. Contin.* **76**, 3097–3112 (2023).

[67] Guo, C., Zhu, J. & Wang, X. MVHS-LSTM: The comprehensive traffic flow prediction based on improved LSTM via multiple variables heuristic selection. *Appl. Sci.*10.3390/app14072959 (2024).

[68] Jiang, J., Han, C., Zhao, W. X. & Wang, J. PDFormer: Propagation delay-aware dynamic long-range transformer for traffic flow prediction. *Proc. AAAI Conf. Artif. Intell.* **37**, 4365–4373 (2023).

[69] Touvron, H. *et al.**Llama 2: Open Foundation and Fine-Tuned Chat Models*. Preprint at 10.48550/arXiv.2307.09288 (2023).

[70] Zhao, W. X. *et al.**A Survey of Large Language Models*. Preprint at 10.48550/arXiv.2303.18223 (2024).

[71] Sennrich, R., Haddow, B. & Birch, A. *Neural Machine Translation of Rare Words with Subword Units*. Preprint at 10.48550/arXiv.1508.07909 (2016).

[72] Vaswani, A. *et al.* Attention is All you Need. in *Advances in Neural Information Processing Systems* vol. 30 (Curran Associates, Inc., 2017).

[73] Lewis, M. *et al.**BART: Denoising Sequence-to-Sequence Pre-training for Natural Language Generation, Translation, and Comprehension*. Preprint at 10.48550/arXiv.1910.13461 (2019).

[74] Raffel, C. *et al.**Exploring the Limits of Transfer Learning with a Unified Text-to-Text Transformer*. Preprint at 10.48550/arXiv.1910.10683 (2023).

[75] Chowdhery, A. *et al.**PaLM: Scaling Language Modeling with Pathways*. Preprint at 10.48550/arXiv.2204.02311 (2022).

[76] Chen, Z. et al. Spatial-temporal short-term traffic flow prediction model based on dynamical-learning graph convolution mechanism. *Inf. Sci.* **611**, 522–539 (2022).

[77] Chen, J. et al. Node connection strength matrix-based graph convolution network for traffic flow prediction. *IEEE Trans. Veh. Technol.* **72**, 12063–12074 (2023).

[78] Gao, H., Jia, H. & Yang, L. An improved CEEMDAN-FE-TCN model for highway traffic flow prediction. *J. Adv. Transp.* **2022**(1), 2265000 (2022).

[79] Huang, X., Tang, J., Yang, X. & Xiong, L. A time-dependent attention convolutional LSTM method for traffic flow prediction. *Appl. Intell.* **52**, 17371–17386 (2022).

[80] Xu, X., Liu, C., Zhao, Y. & Lv, X. Short-term traffic flow prediction based on whale optimization algorithm optimized BiLSTM_Attention. *Concurrency Comput. Pract. Exp.*10.1002/cpe.6782 (2022).

[81] Xu, X., Yang, C., Bilal, M., Li, W. & Wang, H. Computation offloading for energy and delay trade-offs with traffic flow prediction in edge computing-enabled IoV. *IEEE Trans. Intell. Transp. Syst.* **24**, 15613–15623 (2023).

[82] He, R., Xiao, Y., Lu, X., Zhang, S. & Liu, Y. ST-3DGMR: Spatio-temporal 3D grouped multiscale ResNet network for region-based urban traffic flow prediction. *Inf. Sci.* **624**, 68–93 (2023).

[83] Zhou, S. et al. Short-term traffic flow prediction of the smart city using 5G internet of vehicles based on edge computing. *IEEE Trans. Intell. Transp. Syst.* **24**, 2229–2238 (2023).

[84] Naheliya, B., Redhu, P. & Kumar, K. MFOA-Bi-LSTM: An optimized bidirectional long short-term memory model for short-term traffic flow prediction. *Phys. A Stat. Mech. Appl.*10.1016/j.physa.2023.129448 (2024).

[85] Tan, G. et al. A noise-immune and attention-based multi-modal framework for short-term traffic flow forecasting. *Soft Comput.* **28**, 4775–4790 (2024).

[86] Duan, Y. et al. FDSA-STG: Fully dynamic self-attention spatio-temporal graph networks for intelligent traffic flow prediction. *IEEE Trans. Veh. Technol.* **71**, 9250–9260 (2022).

[87] Yan, B., Wang, G., Yu, J., Jin, X. & Zhang, H. Spatial-temporal Chebyshev graph neural network for traffic flow prediction in IoT-based ITS. *IEEE Internet Things J.* **9**, 9266–9279 (2022).

[88] Huo, G. et al. Hierarchical spatio-temporal graph convolutional networks and transformer network for traffic flow forecasting. *IEEE Trans. Intell. Transp. Syst.* **24**, 3855–3867 (2023).

[89] Lai, Q., Tian, J., Wang, W. & Hu, X. Spatial-temporal attention graph convolution network on edge cloud for traffic flow prediction. *IEEE Trans. Intell. Transp. Syst.* **24**, 4565–4576 (2023).

[90] Narmadha, S. & Vijayakumar, V. Spatio-temporal vehicle traffic flow prediction using multivariate CNN and LSTM model. *Mater. Today Proc.* **81**, 826–833 (2023).

[91] Wang, Z., Sun, P., Hu, Y. & Boukerche, A. A novel hybrid method for achieving accurate and timeliness vehicular traffic flow prediction in road networks. *Comput. Commun.* **209**, 378–386 (2023).

[92] Wu, K. et al. Error-distribution-free kernel extreme learning machine for traffic flow forecasting. *Eng. Appl. Artif. Intell.*10.1016/j.engappai.2023.106411 (2023).

[93] Xing, H., Chen, A. & Zhang, X. RL-GCN: Traffic flow prediction based on graph convolution and reinforcement learning for smart cities. *Displays* **1**(80), 102513 (2023).

[94] Yang, D. & Lv, L. A graph deep learning-based fast traffic flow prediction method in urban road networks. *IEEE. Access* **11**, 93754–93763 (2023).

[95] Jia, Q., Zang, J. & Liu, S. Deep learning based traffic flow prediction model on highway research. in (eds. Ghanizadeh, A. & Jia, H.) vol. 13064 (2024).

[96] Lu, W. et al. Traffic flow prediction for highway vehicle detectors through decomposition and machine learning. *Transp. Lett.*10.1080/19427867.2024.2339631 (2024).

[97] Waibel, A., Hanazawa, T., Hinton, G., Shikano, K. & Lang, K. J. Phoneme recognition using time-delay neural networks. *IEEE Trans. Acoust. Speech Signal Process.* **37**, 328–339 (1989).

[98] Hochreiter, S. & Schmidhuber, J. Long short-term memory. *Neural Comput.* **9**, 1735–1780 (1997). 9377276 10.1162/neco.1997.9.8.1735

[99] Hale, T. et al. A global panel database of pandemic policies (Oxford COVID-19 government response tracker). *Nat. Hum. Behav.* **5**, 529–538 (2021). 33686204 10.1038/s41562-021-01079-8

[100] Huang, X., Lan, Y., Ye, Y., Wang, J. & Jiang, Y. Traffic flow prediction based on multi-mode spatial-temporal convolution of mixed hop diffuse ODE. *Electronics*10.3390/electronics11193012 (2022).

[101] Su, Z., Liu, T., Hao, X. & Hu, X. Spatial-temporal graph convolutional networks for traffic flow prediction considering multiple traffic parameters. *J. Supercomput.* **79**, 18293–18312 (2023).

[102] Wang, Z. & Bovik, A. C. Mean squared error: Love it or leave it? A new look at signal fidelity measures. *IEEE Signal Process. Mag.* **26**, 98–117 (2009).

[103] De Gooijer, J. G. & Hyndman, R. J. 25 years of time series forecasting. *Int. J. Forecast.* **22**, 443–473 (2006).

[104] Yi, H. & Bui, K.-H.N. An automated hyperparameter search-based deep learning model for highway traffic prediction. *IEEE Trans. Intell. Transp. Syst.* **22**, 5486–5495 (2021).

[105] *Hyperparameter Tuning for Machine and Deep Learning with R: A Practical Guide*. (Springer Nature, Cham 2023). 10.1007/978-981-19-5170-1.

